# Draft genome sequences of *Lactococcus petauri* isolated from Nile tilapia (*Oreochromis niloticus*) and rainbow trout (*Oncorhynchus mykiss*) in Mexican farms

**DOI:** 10.1128/mra.00301-26

**Published:** 2026-05-18

**Authors:** Eduardo Matínez-Matus, César Ortega-Santana, Ruben Avendaño-Herrera

**Affiliations:** 1 Laboratorio de Patología de Organismos Acuáticos y Biotecnología Acuícola, Facultad de Ciencias de la Vida, Universidad Andrés Bello 28087, Viña del Mar, Chile; 2 Interdisciplinary Center for Aquaculture Research—Applied Research (INCAR2), Universidad Andrés Bello 28087, Viña del Mar, Chile; 3 Actino Lab USM, Centro de Biotecnología DAL, Universidad Técnica Federico Santa María 28090, Valparaíso, Chile; 4 Centro de Investigación y Estudios Avanzados en Salud Animal, Facultad de Medicina Veterinaria y Zootecnia, Universidad Autónoma del Estado de México 27782, Toluca, México; 5 Centro de Investigación Marina Quintay (CIMARQ), Universidad Andrés Bello, Quintay, Chile; University of Maryland School of Medicine, Baltimore, Maryland, USA

**Keywords:** *Lactococcus petauri*, lactococcosis, rainbow trout, Mexican farm

## Abstract

*Lactococcus petauri* is an emerging fish pathogen linked to septicemia. We report draft genome sequences of two *L. petauri* isolates from independent lactococcosis outbreaks in Mexican fish farms. This study confirms *L. petauri* presence in Mexico and provides the first genomic data on this pathogen.

## ANNOUNCEMENT

*Lactococcus petauri* is a gram-positive bacterium first identified in a sugar glider (*Petaurus breviceps*) in the United States (1). Recently, it has been linked to lactococcosis outbreaks in Nile tilapia (*Oreochromis niloticus*) (2) and rainbow trout (*Oncorhynchus mykiss*) across several countries (3). In Mexico, lactococcosis in rainbow trout was previously attributed to *Lactococcus garvieae* (4), with no reports of *L. petauri* until now. Here, we report the draft genome sequences of two *L. petauri* isolates from Mexican outbreaks: Que21-Lp08 from diseased tilapia spleen (Querétaro, 2021) and EP23-Lp16 from rainbow trout kidney (Estado de México, 2023), both confirmed to cause lactococcosis.

Samples for bacterial isolation were aseptically collected from the spleen and kidney of each fish. The samples were streaked onto tryptic soy agar (TSA) and incubated at 25°C for 24–72 h. Resulting pure isolates were stored at −80°C in CryoBank vials (Mast Diagnostica, Reinfeld, Germany). Phenotypically, colonies were small, gram-positive cocci, catalase-negative, and γ-hemolytic.

For genome sequencing, isolates were grown on TSA at 25°C for 24 h. Genomic DNA was extracted using the NucleoSpin Soil kit (MACHEREY-NAGEL, Düren, Germany) and sequenced at SeqCoast Genomics (New Hampshire, USA). Libraries were prepared with the Illumina DNA Prep tagmentation kit and sequenced on an Illumina NextSeq 2000 platform using a 300-cycle flow cell to generate 2 × 150 bp paired-end reads. Raw read quality was assessed using FastQC v0.12.1 (https://www.bioinformatics.babraham.ac.uk/projects/fastqc/).

Sequencing generated 2,615,544 raw reads for Que21-Lp08 and 1,811,876 for EP23-Lp16. Adapters and read ends were trimmed with Trim Galore v0.6.10 (https://github.com/FelixKrueger/TrimGalore), and coverage was normalized by down-sampling reads with the BBNorm function implemented in BBTools v39.73 (5). Genome assemblies were generated using Unicycler v0.4.8 (6) and evaluated with QUAST v5.2.0 (7). Genome completeness and contamination were assessed using CheckM v1.2.4 (8), and functional annotation was performed using the NCBI Prokaryotic Genome Annotation Pipeline (PGAP) v6.10 (20 January 2026) (9). Antimicrobial resistance genes were identified using the Resistance Gene Identifier in the Comprehensive Antibiotic Resistance Database (https://card.mcmaster.ca/analyze/rgi). All software was executed with default parameters.

The draft genome assemblies (Table 1) comprised 10 (Que21-Lp08) and 9 (EP23-Lp16) contigs, with total lengths of 2,028,205 and 2,028,373 bp, respectively. Both genomes had a G + C content of 38.08%. The N50 values were 1,677,321 bp for Que21-Lp08 and 1,677,957 bp for EP23-Lp16, with average sequencing coverages of 187× and 130×, respectively. Genome annotation was performed using the automated NCBI PGAP with default parameters. A total of 1,995 genes, including 1,921 CDSs, 46 tRNAs, and 6 rRNAs, were predicted in Que21-Lp08, whereas 1,999 genes, 1,923 CDSs, 48 tRNAs, and 6 rRNAs were predicted in EP23-Lp16.

**TABLE 1:** Assembly statistics and accession numbers of *Lactococcus petauri* strains isolated in Mexico and deposited in the NCBI database

Strain	SRA	Accession no.	No. of reads^*a**,**b*^	Total length ^*c*^	Completeness^*d*^	Genome coverage	Contamination (%)^*d*^	Contigs^*c*^	%GC^*c*^	N50 (bp)^*c*^	Genes^*e*^	CDS^*e*^
Que21-Lp08	SRR36865524	JBTTGY000000000	2,615,544/2,564,030	2,028,205	98.6	187×	1.4	10	38.08	1,677,321	1,995	1,921
EP23-Lp16	SRR36865523	JBTTHV000000000	1,811,876/1,782,122	2,028,373	98.6	130×	1.4	9	38.08	1,677,957	1,999	1,923

^*a*^ Original read number.

^*b*^ Normalized read depth using BBNorm.

^*c*^ Determined using QUAST v5.2.0.

^*d*^ Determined by CheckM v1.2.4.

^*e*^ Determined using PGAP v6.10.

Species identification was confirmed using the Type (Strain) Genome Server (10) and JSpeciesWS–Taxonomic Thresholds (11). Both isolates clustered with the *L. petauri* 159469ᵀ (accession no. GCF_002154895.1), with a digital DNA–DNA hybridization value of 86%. Average nucleotide identity (12) relative to *L. petauri* 159,469ᵀ was 98.31% for Que21-Lp08 and 98.34% for EP23-Lp16, confirming their taxonomic assignment. The *lsaD* antimicrobial resistance gene was detected in both genomes.

These genomes represent the first *L. petauri* sequences from Mexican aquaculture and provide a resource for studies on genomic diversity and molecular diagnosis of lactococcosis in fish.

## Data Availability

This whole-genome shotgun project has been deposited in DDBJ/ENA/GenBank under the accession numbers JBTTGY000000000 (Que21-Lp08) and JBTTHV000000000 (EP23-Lp16). BioProject ID PRJNA1403790 is publicly available and contains BioSample SAMN54690575 and SAMN54691117. The corresponding SRA records are SRR36865524 (Que21-Lp08) and SRR36865523 (EP23-Lp16).

## References

[1] Goodman LB, Lawton MR, Franklin-Guild RJ, Anderson RR, Schaan L, Thachil AJ, Wiedmann M, Miller CB, Alcaine SD, Kovac J. 2017. Lactococcus petauri sp. nov., isolated from an abscess of a sugar glider. Int J Syst Evol Microbiol 67:4397–4404. doi:10.1099/ijsem.0.002303 28945531 PMC5845659

[2] Egger RC, Rosa JCC, Resende LFL, de Pádua SB, de Oliveira Barbosa F, Zerbini MT, Tavares GC, Figueiredo HCP. 2023. Emerging fish pathogens Lactococcus petauri and L. garvieae in Nile tilapia (Oreochromis niloticus) farmed in Brazil. Aquaculture 565:739093. doi:10.1016/j.aquaculture.2022.739093

[3] Vela AI, del Mar Blanco M, Colussi S, Kotzamanidis C, Prearo M, Altinok I, Acutis PL, Volpatti D, Alba P, Feltrin F, Ianzano A, Domínguez L, Fernández-Garayzábal JF. 2024. The association of Lactococcus petauri with lactococcosis is older than expected. Aquaculture 578:740057. doi:10.1016/j.aquaculture.2023.740057

[4] Ortega C, Irgang R, Valladares-Carranza B, Collarte C, Avendaño-Herrera R. 2020. First identification and characterization of Lactococcus garvieae isolated from rainbow trout (Oncorhynchus mykiss) cultured in Mexico. Animals (Basel) 10:1609. doi:10.3390/ani10091609 32916954 PMC7552202

[5] Bushnell B. 2014. BBMap: a fast, accurate, splice-aware aligner. https://github.com/bbushnell/BBTools.

[6] Wick RR, Judd LM, Gorrie CL, Holt KE. 2017. Unicycler: resolving bacterial genome assemblies from short and long sequencing reads. PLoS Comput Biol 13:e1005595. doi:10.1371/journal.pcbi.1005595 28594827 PMC5481147

[7] Gurevich A, Saveliev V, Vyahhi N, Tesler G. 2013. QUAST: quality assessment tool for genome assemblies. Bioinformatics 29:1072–1075. doi:10.1093/bioinformatics/btt086 23422339 PMC3624806

[8] Parks DH, Imelfort M, Skennerton CT, Hugenholtz P, Tyson GW. 2015. CheckM: assessing the quality of microbial genomes recovered from isolates, single cells, and metagenomes. Genome Res 25:1043–1055. doi:10.1101/gr.186072.114 25977477 PMC4484387

[9] Tatusova T, DiCuccio M, Badretdin A, Chetvernin V, Nawrocki EP, Zaslavsky L, Lomsadze A, Pruitt KD, Borodovsky M, Ostell J. 2016. NCBI prokaryotic genome annotation pipeline. Nucleic Acids Res 44:6614–6624. doi:10.1093/nar/gkw569 27342282 PMC5001611

[10] Meier-Kolthoff JP, Göker M. 2019. TYGS is an automated high-throughput platform for state-of-the-art genome-based taxonomy. Nat Commun 10:2182. doi:10.1038/s41467-019-10210-3 31097708 PMC6522516

[11] Richter M, Rosselló-Móra R, Oliver Glöckner F, Peplies J. 2016. JSpeciesWS: a web server for prokaryotic species circumscription based on pairwise genome comparison. Bioinformatics 32:929–931. doi:10.1093/bioinformatics/btv681 26576653 PMC5939971

[12] Richter M, Rosselló-Móra R. 2009. Shifting the genomic gold standard for the prokaryotic species definition. Proc Natl Acad Sci USA 106:19126–19131. doi:10.1073/pnas.0906412106 19855009 PMC2776425

